# Factors affecting child welfare and protection workers' intention to quit: a cross-sectional study from Norway

**DOI:** 10.1186/s12960-023-00829-1

**Published:** 2023-06-05

**Authors:** Kristel Høie Nilsen, Camilla Lauritzen, Svein Arild Vis, Anita Iversen

**Affiliations:** 1 RKBU Institute, UiT The Arctic University of Norway, Langnes, PO Box 6050, 9037 Tromsø, Norway; 2 Centre for Faculty Development, Faculty of Health Sciences, UiT The Arctic University of Norway, Tromsø, Norway

**Keywords:** Child welfare, Child advocacy, Burnout, Intention, Personnel turnover

## Abstract

**Introduction:**

High turnover rates have been a problem for Norwegian child welfare and protection services for years. The main aim of this study was to identify which factors affect Norwegian child welfare and protection (CWP) workers intention to quit their job and whether there is a difference between experienced (< 3 years) and less experienced workers.

**Methods:**

A cross-sectional survey was performed among 225 Norwegian child welfare and protection workers. Data were collected using a self-report questionnaire. Turnover intention was examined using a variety of job demands and resources as possible predictors. *T* tests were used to study mean differences in variable scores between experienced and less experienced workers and linear regression analysis was employed determining predictors of intention to quit.

**Results:**

For the total sample (*N* = 225) the most important predictors for intention to quit were workload, burnout, engagement, and views on leadership. Higher emotional exhaustion and cynicism, and low professional efficacy predicted a higher score on the intention to quit scale. High engagement and leadership satisfaction predicted lower scores. The effect of workload was moderated, such that intention to quit among less experienced workers increased more with high workload than it did among more experienced child welfare workers.

**Conclusions:**

The conclusions are that job demands affect experienced and less experienced CWP workers differently and that when designing preventive efforts to reduce turnover this must be considered.

## Background

The turnover of the child welfare and protection (CWP) workforce has been extensively studied for decades but is still an area of concern due to persistently high rates [1–7]. Continually having to train new employees affects the organization negatively and affects the quality of services delivered [7–12]. High turnover rates directly interfere with the important task of developing and maintaining good and lasting relationships with the children and families in need of assistance from CWP services. For children, turnover can present a traumatic loss when the worker they have come to trust and rely on quits [10, 13].

The Norwegian child welfare system consists of two parts; the municipal child protective agencies who are responsible for conducting investigation, provision of home-based services and foster homes, and state-run services for out of home placement in institutions. A total of 8500 employees are responsible for the day-to-day work that follows from the child welfare act in Norway. Norwegian child welfare and protection services are organized in a way that makes each individual employee, by themselves or in pairs, responsible for a number of cases. Cases are discussed in teams or with superiors if necessary. Each individual caseworker’s competence is, therefore, essential to correctly identify and ensure the child’s needs [14].

Norwegian child welfare and protection workers have the highest turnover rates in the municipal sector at 20.9%, compared to elementary schools at 12.8%, and 15.2% for kindergartens [15]. A comparable situation exists in several countries worldwide with turnover rates ranging from 23 to 60% [3, 10, 16–19]. The global high rate of turnover is indicative of the shared commonalities of CWP work, across different nations’ legislation and judicial frameworks for organizing the CWP work [20–22].

Typically, it takes about 2 years for a new child welfare employee to learn what needs to be done in their jobs and to develop the knowledge, skills, abilities, and dispositions to work independently [23, 24]. Previous research shows that intention to quit is higher among the newly employed and decreases as tenure increases [16, 25]. This is to be expected, since workers with a low intention to quit are more likely to remain in their position as time goes by. It does not mean that more experienced workers do not quit, although it is possible that they quit for other reasons. However, there are few studies investigating differences between less experienced and more experienced workers in what affects their intention to quit. The current study investigates previously found factors affecting Child Welfare and Protection (CWP) workers' intention to quit their job and adds to this knowledge by investigating whether there are differences in what affects the intention to quit for newly employed compared to more experienced workers. This may lead to the development of more targeted measures for staff retention.

### Intention to quit and factors affecting this

The study of intention to quit is highly relevant, because previous research has identified a strong correlation between behavioral intention and actual behavior [26–30], and data from several studies suggest that intention to quit is the best predictor of actual turnover [28, 30–32]. More specifically, Tett and Meyer [33] found a moderate to strong correlation (*r* = 0.45) between intention to quit and actual turnover. Turnover intention may be a better measure of a challenging workplace environment than actual turnover, because turnover behavior is contingent on many external factors, such as perceived and actual alternatives, and monetary interests [17, 18].

For the CWP workforce turnover has previously been connected to the nature of the work combined with organizational factors, such as high workloads, work–family disbalance, staff shortage, time pressure, and emotionally demanding tasks [34, 35]. CWP workers in general have more than double the risk of experiencing high emotional demands and role conflict in their work, and almost six times the risk of experiencing violence or threats of violence, compared to the general workforce [36]. They also have an increased risk of experiencing sleep problems, psychological distress, and exhaustion [12, 36–40].

Burnout has been shown to significantly affect the intention to quit and actual turnover among CWP professionals [38, 39, 41–46]. Previously identified factors that contribute to the development of burnout are overwhelming job demands, high workloads, low job control, threats of violence, and working with people who have experienced trauma or stressful life events [47–50]. These factors are all familiar challenges in CWP work.

In a 2017 study on secondary traumatic stress, burnout, and compassion satisfaction among Norwegian child protection workers, the results showed that increased workload, work–family conflict, role conflict, and a decrease in support from superiors and co-workers all increased burnout [38]. Hence, these factors might be important predictors of intention to quit as well.

CWP workers experience particularly poor working conditions in this regard due to excessive paperwork, long working hours, ineffective bureaucratic structures, and little opportunity for advancement [51, 52]. Leadership plays a key role in managing the work environment and hence affects how workers perceive their working conditions and their job satisfaction [4, 53], and studies show that child welfare managers have particularly demanding and challenging leadership tasks [12, 54].

Despite this high prevalence of psychological and emotional strain, CWP workers report that their work is gratifying and important [55, 56]. CWP-workers’ idealism and purpose for choosing to work with families and children is a source of motivation, compassion satisfaction, and engagement when they feel that their help is making a difference [57, 58]. Engagement is a dynamic and temporary state of inner motivation nurturing growth, learning and development, and fosters organizational commitment [59, 60]. Engagement and organizational commitment play a key role in turnover [4, 35].

### Framework for the current study

In the current study, the organizational outcome turnover intention is examined using a variety of job demands and resources as predictors, inspired by the Job Demands-Resources (JD-R) model [41]. According to the JD-R model, job characteristics can be categorized as either a job demand or a job resource, and it is the discrepancy between job demands and job resources that may lead to burnout and turnover [41]. Job demands are defined as “physical, social or organizational aspects of the job that require sustained physical or mental effort and are, therefore, associated with physiological and psychological costs” [41], whereas job resources are defined as “physical, psychological, social or organizational aspects of the job that may do any of the following (a) be functional in achieving work goals (b) reduce job demands at the associated physiological or psychological costs (c) stimulate personal growth and development” [41]. The model has been used extensively in studies worldwide evaluating work-related outcomes and workers’ well-being, and focuses on stress factors, as well as job satisfaction and engagement as their positive counterparts. This study does not test the JD-R model but utilizes the theory behind it in the selection of predictors.

## Methods

### Aims of the study

The aims of the current study are to (a) explore the characteristics of Norwegian CWP workers in terms of job resources, job demands, workload, burnout, and intention to quit, (b) determine predictors of CWP workers’ intention to quit, and (c) determine whether worker status as less experienced versus tenured moderates the effects of predictors of intention to quit.

### Participants and design

The study was designed as a cross-sectional study. Participants were CWP workers from 80 different municipalities distributed across Norway (*N* = 225) who were employed in either municipal or state child welfare. The sample was analyzed as a whole and subdivided into two groups of less experienced and experienced workers. The participants designated as experienced in this study are CWP workers who have more than 3 year experience in child welfare work. All participating CWP workers gave informed consent, and the study was approved by the Norwegian Centre for Research Data (NSD). A paper questionnaire was distributed by the organizers of the study to the experienced workers at a postgraduate gathering, whereas the less experienced workers were given their questionnaire by an experienced worker when they returned from the gathering. 291 questionnaires were handed out, and 225 were returned, giving a total response rate of 77% (88% for experienced CWP workers and 63% for less experienced CWP workers).

For descriptive details, see Table 1.

**Table 1:** Descriptive statistics, *n* = 225

	*n*	*%*
Female	198	89.2
Male	24	10.8
Age	222	
30 or younger	62	27.9
31–40	85	38.3
41–50	56	25.2
50 or older	19	8.6
Married or cohabitating	222	
Yes	175	78.8
No	47	21.2
Highest educational level	220	
Primary school	1	0.4
College or University 1–3 years	120	54.5
College or University 4 < years	99	45
Type of education	210	
Child welfare education	129	61.4
Other	81	38.6
Employment	222	
Full time	205	92.3
Part time	13	5.9
Sick leave (ordained by a physician)	4	1.8
Receives supervision	222	
Yes	160	72.1
No	62	27.9

### Measures

A questionnaire comprising 179 questions was used to collect information, including both demographic items and measures assessing job satisfaction, job engagement, leadership satisfaction, social support, burnout, and intention to quit. The different measures used are further described below.

#### Demographic variables

Questions such as age group, gender, education, and municipality were used to gather information for demographic variables. We also asked how many years they had worked in child welfare. Since new CWP workers often start work directly after completing their studies, we wanted to check whether age or years of experience in their current profession affected the results. Whether the CWP worker received systematic guidance and if so, what kind, was also asked about.

#### Job demands

For this study, the following demands were examined: workload, work conflict, and work–family conflict.

Nine items from the Total Workload Questionnaire (TWQ) [61] were used to obtain factors related to workload, including questions such as “To what extent do you feel you have enough time to perform your work duties?” and “Do you feel you have too much to do?”. The items were answered on a seven-point Likert scale. Cronbach’s alpha was 0.83 for workload.

Four items were used to measure participants' feelings about work conflicts (two items) and work–family conflict (two items) including questions such as “I often experience conflict with other colleagues at work” and “I often feel conflict between my work and my family roles or other obligations” [62]. Cronbach’s alpha was 0.65 and 0.86, respectively.

#### Job resources

Aspects included in this study were autonomy, social support, and leadership satisfaction.

Seven items were used to assess autonomy obtained from the Total Workload Questionnaire, including questions such as “To what extent do you have direct influence on what you do in your job?” and “To what extent can you, on your own initiative, realize your own ideas in your job?” [61, 63]. Cronbach's alpha was 0.72.

Social support was measured using eight items, including questions such as “Your co-workers show you warmth and kindness when you are facing problems at work”, and “Your boss assists you in completing a difficult task” [64]. Cronbach’s alpha was 0.83 for social support.

Seven items from the Leadership scale developed by Shipton, Armstrong, West, and Dawson [65] were used to measure participants' leadership satisfaction. A five-point Likert scale was used, ranging from (1) Not at all to (5) To a very large extent describing participants’ line managers with items such as “Clearly describes the goals of the service for employees” and “Takes account of both service requirements and staff needs when implementing changes”. Cronbach’s alpha for this scale was 0.88.

#### Engagement

To assess job engagement, the Norwegian short version of the Utrecht work engagement scale [66] consisting of nine items was used. Statements such as “I feel strong and energetic at work”, “My work inspires me” and “I look forward to going to work when I wake up in the morning” were rated from (0) Never in the past 6 months to (6) Daily. We used the scale as one-factor and calculated the total scale scores [67]. Cronbach's alpha was 0.91.

#### Job satisfaction

Four items were used to assess job satisfaction, all obtained from the Total Workload Questionnaire [61, 63]. Questions such as “How interesting do you find your work?” and “In total, how well do you feel you are able to deal with problems arising at work?” were used. Cronbach's alpha was 0.79.

#### Burnout

The Norwegian version of the Maslach burnout inventory [68] consisting of 16 items measuring emotional exhaustion, professional efficacy, and cynicism, was used to assess burnout, with five items measuring emotional exhaustion, six items measuring professional efficacy and five items measuring cynicism. Our sample has a Cronbach's alpha of 0.89, 0.84 and 0.78, for emotional exhaustion, professional efficacy, and cynicism, respectively.

#### Intention to quit

A scale consisting of five items was used in this study [69]. Items such as “I often think about quitting my job” and “I will probably actively look for a new job within the following year” were rated using a five-point scale ranging from (1) Strongly disagree to (5) Strongly agree. In our sample Cronbach’s alpha for the scale was 0.88.

#### Reliability of measures

The overall reliability for the scales used in this study was good. Alpha values between 0.6 and 0.7 are viewed as acceptable and values between 0.8 and 0.95 are considered very good [70].

### Statistical analysis

All analyses calculating both descriptive statistics and regression diagnostics were conducted using IBM SPSS Statistics, version 26. Both job demands and job resources were coded so that a higher score indicates higher job demands or resources. The scale scores are used for *t* test and regression analysis, whereas some items are presented with percentages for descriptive purposes.

*T* tests were conducted to examine differences in mean scores between less experienced and experienced workers.

Investigations of correlations among predictors, and the variation inflation factor values, indicated that there were no issues with multicollinearity in the data set.

Following the recommendations for model building through purposeful selection of variables set out by Hosmer, Lemeshow and Sturdivant [71], a series of regression analyses was conducted. First, the simplest relationship between each of the predictors and intention to quit was examined. Second, interactions between work experience and each of the predictors were assessed. Third, a full multi-variable model was estimated using only the predictors and interaction terms that were associated with intention to quit in the simple analysis (*p* < 0.25). Fourth, a parsimonious model was estimated by stepwise elimination of variables from the full model that did not meet significance level of *p* < 5%. When removing variables, the remaining beta values were checked to see if they changed substantially (> 20%), which could indicate that the removed variable had a confounding effect and should be kept in the model despite having a higher *p* value. No such confounders were identified.

## Results

### Job characteristics of CWP workers

The mean number of years in the current profession for the total sample was 6.38 years (SD = 5.69), with a range of 24 years. The mean number of years for less experienced workers was 1.63 years (SD = 2.75), while for experienced workers, the mean was 9.6 years (SD = 4.62). Mean scores for predictor variables are shown in Table 2.

**Table 2:** *T* test for differences in predictor mean scores for less experienced and experienced CWP-workers

	Total sample		Less experienced		Experienced		Group difference
	(*n* = 221–225)		(*n* = 94)		(*n* = 127)		
	*M*	SD	*M*	SD	*M*	SD	*t*
Intention to quit	1.910	0.941	2.036	1.165	1.816	0.764	1.596
Job demands							
Work load	4.553	0.995	4.324	1.071	4.724	0.936	2.974**
Work conflict	1.571	0.792	1.345	0.595	1.709	0.865	3.736***
Work–family conflict	3.341	1.557	3.244	1.587	3.465	1.559	1.040
Job resources							
Autonomy	5.104	0.667	4.936	0.776	5.228	0.623	3.013**
Social support	3.306	0.504	3.381	0.507	3.249	0.494	− 1.953
Leadership	3.663	0.640	3.776	0.634	3.588	0.626	2.178*
Engagement	4.358	0.887	4.375	0.985	4.349	0.877	− 0.213
Job satisfaction	5.618	0.717	5.460	0.946	5.822	1.199	2.436*
Burn out							
Emotional exhaustion	1.686	1.206	1.766	1.463	1.640	1.035	− 0.714
Professional efficacy	4.457	0.866	4.235	0.971	4.608	0.799	3.154**
Cynicism	0.889	0.914	1.012	1.074	0.811	0.799	1.531

Significant group differences *p < 0.05 **p < 0.01 ***p < 0.001 (two-tailed)

72.1% of the participants received guidance. The majority indicated that directly case-related supervision was the most usual form of guidance, while debriefing and reflective supervision with the aim of professional development was less common.

52.2% of the participants stated that a reduced workload was desirable, and 69.8% indicated that they wanted more time to spend with each child/young person/family to a large/very large degree.

89.6% of the participants gave answers indicating that they feel enthusiastic about their work on a weekly to daily basis, and 82.9% stated that they feel full of energy in their work on a weekly to daily basis. 84.2% stated that they look forward to going to work when they wake up in the morning.

In this sample 15.6% agreed partly or completely with statements indicating that they were looking for work elsewhere. For a statement about actively looking for work elsewhere within the next three years 22.9% answered that they agreed or strongly agreed.

### Predicting intention to quit

Of the 15 possible predictors 12 were significant in bivariate analyses. In the multi-variable analyses, five predictors and one interaction term remained significant. For the total sample, the most important predictors of intention to quit were engagement, emotional exhaustion, cynicism, professional efficacy, and leadership satisfaction. High cynicism and emotional exhaustion, low professional efficacy and leadership satisfaction predicted higher chances of an intention to quit, and high engagement predicted lower chances of an intention to quit.

Significant interactions were found between experience and workload, engagement, and emotional exhaustion in the bivariate analysis. In the parsimonious model, interaction between job experience and workload remained a significant predictor of intention to quit. This model explained 45.6% of the variation in intention to quit (Table 3).

**Table 3:** Regression analyses of variables predictive value on dependent variable "Intention to quit"

Variables	Total sample Bivariate				Full model		Parcimonius model	
	*t*	*p*	Beta	ci	Beta	*p*	Beta	*p*
Job demands								
Work load	4.97	< 0.001	0.300	0.181–0.419	− 0.037	0.706	− 0.073	0.364
Work conflict	1.269	0.206	0.103	− 0.057– 0.264				
Work–family conflict	3.064	0.002	0.124	0.044 – 0.204	0.001	0.980		
Job resources								
Autonomy	− 5.499	< 0.001	− 0.469	− 0.638 – 0.301	0.034	0.714		
Social support	− 3.890	< 0.001	− 0.481	− 0.724 − 0.237	− 0.109	0.397		
Leadership satisfaction	− 3.314	0.001	− 0.330	− 0.527 − 0.134	− 0.148	0.125	− 0.186	0.025
Engagement	− 8.597	< 0.001	− 0.581	− 0.637 − 0.400	− 0.393	< 0.001	− 0.437	< 0.001
Job satisfaction	− 5.257	< 0.001	− 0.285	− 0.393 − 0.178	− 0.036	0.497		
Burn out								
Emotional exhaustion	8.491	< 0.001	0.384	0.295 – 0.474	0.088	0.337	0.136	0.029
Professional efficacy	− 3.838	< 0.001	− 0.266	− 0.403– 0.129	0.190	0.020	0.166	0.028
Cynicism	7.836	< 0.001	0.482	0.360 – 0.603	0.174	0.013	0.197	0.004
Experienced/less experienced	1.702	0.090	0.220	− 0.035 – 0.474	− 0.176	0.837	− 0.842	0.076
Age	− 2.621	0.009	− 0.178	− .312 − 0.044	0.067	0.347		
Interactions with experience								
Work load	2.828	0.005	0.337	0.102 – 0.572	0.171	0.209	0.246	0.017
Work conflict	0.257	0.798	0.049	0.328 – − 0.426				
Work–family conflict	0.727	0.468	0.059	− 0.101– 0.219				
Autonomy	− 1.807	0.72	− 0.314	− 0.657–0.028				
Social support	− 0.911	0.363	− 0.227	− 0.719–0.264				
Leadership satisfaction	0.690	0.491	0.140	− 0.259–0.539				
Engagement	− 2.994	0.003	− 0.353	− 0.585–− 0.121	− 0.087	0.502		
Job satisfaction	− 6.094	< 0.001	− 0.673	− 0.891–0.456				
Emotional exhaustion	2.657	0.008	0.241	0.062–0.420	0.067	0.577		
Cynicism	1.414	0.159	0.176	− 0.069–0.421				
Professional efficacy	− 0.686	0.493	− 0.098	− 0.378–0.183				

### Examining group differences between less experienced and experienced workers

There were differences in some but not all the proposed predictors. Most notably, less experienced workers scored lower on job satisfaction and professional efficacy, and higher on leadership satisfaction and intention to quit. Experienced workers scored higher on work conflict and autonomy (Table 2).

In the bivariate analyses, several of the predictor variables were significantly associated with ITQ. In the final multivariable model, all three dimensions of the burnout inventory emerged as significant predictors of ITQ. The group difference for the variable professional efficacy between less experienced and experienced workers showed that the less experienced had a lower mean.

We did not find any evidence that work experience moderated the predictor effect of any of the measures of burn out or job resources. There was, however, a significant interaction effect between workload and work experience.

**Fig. 1:**
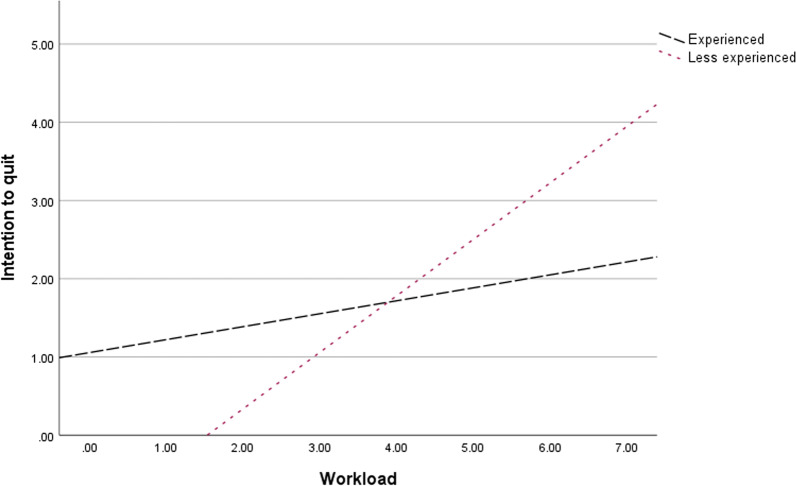
How workload affects experienced and less experienced CWP workers’ intention to quit differently

A plot of predicted values for intention to quit for different values of workload among less experienced and experienced workers is shown in Fig. 1. This shows that at zero workload, intention to quit is lower for less experienced than for experienced workers. When workload increases; however, intention to quit increases faster for less experienced than for experienced workers.

## Discussion

### Job characteristics of CWP workers

20% of the participants stated that they had too much to do to a large or very large extent. These findings are consistent with findings from the SKO study [46, 72] and several other studies of workload in child protection services [16, 19, 25, 73]. Yet, workload sizes have been found to be smaller for child welfare workers than for mental health professionals and for family service agencies but are still deemed unmanageable due to the complexities involved in each case [19].

It is customary to shield new and less experienced workers from the number and complexity of cases. The similarity in the number of hours spent working per week might indicate that this is not practiced, or that they are shielded, but not sufficiently. It could also be that, because they are new, they spend more time on a smaller number of tasks than experienced workers. Since high workloads affect the intention to quit for less experienced more strongly than they affect experienced workers, this should be investigated further.

### Predicting intention to quit

All three dimensions of burnout emerged as predictors of intention to quit. Previous research has shown the connection between burnout and intention to quit, especially for the dimension of emotional exhaustion [3, 19, 45, 46]. Emotional exhaustion is the most prevalent factor of burnout and is often associated with absenteeism. However, cynicism is more closely related to turnover intention. Cynicism is connected to a lack of job resources, whereas exhaustion stems from overwhelming job demands. The mean of emotional exhaustion for this sample (1.69, SD 1.23) is similar to previous findings of emotional exhaustion (*M* 1.71, SD 1.25) in healthcare professionals working in public health care in Norway [46].

Other studies have often focused mainly on exhaustion and have not included cynicism and professional efficacy in their analyses [46, 55, 72]. Having included all three dimensions in our analysis, this study also found that professional efficacy and cynicism predicted intention to quit. This may be because CWP workers who realize they have become cynical or distant also realize that this will affect the quality of their work and do not want to be part of providing sub-optimal services. It is also possible that being compassionate, and caring are such important parts of the CWP worker ethos that cynicism may cause CWP workers to question their professional work identity in a manner that is different from other professions. Hence, cynicism may be especially damaging for CWP workers’ work retention. Elevated levels of cynicism might affect the quality of services provided to children and families in a negative way through workers distancing themselves from their clients or being less focused on finding flexible solutions to problems.

Leadership satisfaction as a predictor of intention to quit is known from previous studies [4, 53, 74, 75]. The results of this study, which corroborate these previous findings, are important in the ongoing attempts to increase competence and retention. In CWP work, leadership is of particular interest, because it encompasses several distinct types of management: child welfare management, professional management, strategic management, personnel management, financial management and public management (The Norwegian Directorate for Children; Youth and Family Affairs, 2017). Combining classic leadership functions, such as budgeting and employee management, and also professional responsibility for decision-making in complex child welfare issues might lead to conflicting interests [54]. The managers need to make decisions in accordance with the service’s strategic, professional, and values-based platform. Balancing these sometimes-contradictory demands might be hard to understand for CWP workers without management responsibilities, and hence affect their satisfaction with their manager.

In our study 15.6% of CWP workers agreed partly or completely with statements regarding intentions to quit and that they were looking for work elsewhere within the following year, and 22.9% within 3 years. This is low compared to national surveys conducted in 2019 and 2021, where 21% of CWP workers stated that they wanted to look for work outside child welfare and protection services [76]. Previous research shows that intention to quit is higher among the less experienced workers, and decreases as tenure increases [45]; hence, the higher proportion of experienced workers in this study could affect the mean.

Of the five significant predictors, engagement had the strongest effect on intention to quit. This is consistent with previous research [6, 77]. Engagement is viewed as the counterpoint to burnout; thus, improving engagement can lower burnout level and reduce turnover intention. It is subsequently of great interest to identify what predicts engagement for CWP workers so that more precise efforts can be made to improve retention.

### Examining group differences between less experienced and experienced workers

We found some interesting differences between less experienced CWP workers and experienced CWP workers (Table 2). Work conflict scores were higher among the experienced workers. One explanation for this may be that experienced workers with more responsibility have more conflicts related to disagreements in case management. Disagreements may be perceived as interesting and refreshing if you are not the case manager responsible. However, if you are responsible for reconciling differences and providing the ultimate recommendation for action, it may more likely be perceived as a problem and a potential source of workplace conflict. The same explanation may apply to the more positive view on leadership exhibited by the less experienced. As a less experienced worker, you may not have as much direct case-management contact with your line manager and, therefore, may have fewer arguments, hence a more positive view.

It is not surprising that experienced workers felt more autonomy compared to the less experienced, but there can be several explanations for it. One explanation might be that more experience results in more responsibility, and more trust and freedom to solve tasks as you see fit, hence giving the employee a sense of autonomy. Another explanation might be that less experienced workers with the same amount of responsibility and freedom related to their tasks do not perceive this as autonomy but rather as excessive workload, insecurity, and stress, affecting their professional efficacy and job satisfaction.

The less experienced workers scored significantly lower on job satisfaction. We believe this is, at least in part, caused by feeling new, overwhelmed, and not having settled in yet. Another contributor could be that the less experienced scored lower on professional efficacy. Professional efficacy is a measure of how well you feel you are mastering your work. Being less experienced or new, often having just finished your studies, means you have to transform your theoretical knowledge into practical knowledge, while at the same time, you have to learn everything a new worker must learn, such as routines and regulations of the workplace, who is who when you need help, new data software and so on. In addition, autonomy and job satisfaction are linked, with higher degrees of autonomy leading to higher degrees of job satisfaction [78].

Because we also found differences among less experienced and experienced workers in many of the variables assumed to be predictors of intention to quit, we had to ask ourselves if job experience has a moderating effect on predictors of intention to quit. In other words, do less experienced and experienced workers want to quit their jobs for diverse reasons? This is an interesting and important question to address, and one that has practical implications for how to prevent less experienced workers from leaving the CWP service. We, therefore, found it crucial to investigate potential interaction effects among job experience and predictors of intention to quit when developing the multivariable prediction model. We found significant interactions between experience and workload, engagement, job satisfaction, and emotional exhaustion in the bivariate analyses; however, only workload stayed significant in the multivariable analyses. The interaction effect between workload and experience showed that intention to quit for less experienced workers was more affected by high workloads than intention to quit for experienced workers. This indicates that high workloads have a more detrimental effect on job retention among the less experienced than among more experienced workers. This is in accordance with findings from a study from 2019 on early departure among child welfare workers [25]. One explanation for this might be that higher workloads contribute to higher emotional demands. For the less experienced workers this might be more overwhelming, and because they do not have the experience that tells them that it is “normal” to be emotionally affected, or how to prioritize tasks to minimize stress, they are dependent on co-workers and supervisors noticing this and guiding them through it. In a high-paced work situation where you never have enough time, it might be challenging to ask for help or to identify what your co-worker is struggling with. The less experienced might also want to appear to be on top of their tasks, making them more reluctant to ask for help or admitting that they cannot take on any more cases.

The indication that high workloads affect intention to quit more strongly among less experienced CWP workers might also lend support to the notion that the new and less experienced workers experience a shock when they cross over from theory to practice, which leads them feeling overwhelmed and exhausted and seeking work elsewhere [25, 79–81]. This “shock”, stemming from the discrepancy between educational knowledge and practical competence, is recognized in several comparable professions, such as nursing and teaching [82].

An attempt to guide the newly employed through their first year by offering reflective supervision has been debated extensively without clear results for the child welfare field. The Norwegian government aimed to implement a supervised first year as standard for newly educated CWP workers as part of their attempts to strengthen the municipal CWP services [83]. A study from 2015 found that this initiative had not worked as intended due to differences in the understanding of what reflective supervision entails, lack of competence among supervisors, and lack of resources to make reflective supervision a permanent measure within the agency [84]. The current study’s interaction analyses showed that the intention to quit among the less experienced appears to be more affected by high job demands, and that continued efforts to support newly employed and less experienced workers should be put forward. This will be an important aspect to consider when planning and budgeting for the implementation of the child welfare reform of 2022 [85]. The reform aims to provide better services for children and families in need by providing a more holistic and preventive child welfare service. Results from this study show that special support for all new employees in CWP services would be beneficial.

### Limitations and future directions

The sample for this study may not be a representative sample of the child welfare and protection workforce due to convenience sampling. All the experienced workers had chosen to attend a post-graduate event, indicating that they might be more motivated and have a lower intention to quit than experienced workers who are not attending any educational program. Therefore, some selection bias is to be expected.

The difference in response rate between experienced less experienced workers may be a result of the fact that the experienced CWP workers filled out and returned the questionnaire on site, whereas less experienced workers had to return their questionnaires by mail to the research coordinator. However, the response rate is still relatively high, with 66% for the group responding by mail and 88% for those filling out and returning their questionnaires on site and allows for valid interpretations of the results.

Our chosen limit of 3 years to separate less experienced and experienced workers could also have been set at 2 years or 1 year, depending on how you want to define "experienced". However, our choice is guided by consensus on how long it takes to acquire the knowledge and competence required to perform the job independently (minimum 2 years), and the number of years of experience in child protection required to participate in the postgraduate program (3 years) y[23, 24].

Differences in working conditions across countries, and diverse ways of organizing child welfare and protection services, might limit the generalizability to other countries with a significantly different approach in this regard.

This study contributes to the field by providing a unique insight into significant differences in what affects intention to quit among less experienced and more experienced CWP workers and shows that future research should consider that predictors might be different for less experienced and more experienced CWP workers. It suggests it will be important to distinguish between efforts made to improve retention among these groups. Since the predictors affect less experienced and experienced workers differently, it may be the case that what influences the predictors also differentiates. Future research should aim to explore whether engagement and burnout have the same predictors of less experienced as for more experienced CWP workers.

## Conclusion and practical implications

The results showed that 15.6% of all participants indicated they had intentions to quit their job. Low work engagement, professional efficacy leadership satisfaction, and high emotional exhaustion, cynicism and workload were the best predictors of intention to quit, with interactions showing that both job demands and resources affected the intention to quit for the less experienced to a greater extent than experienced workers’ intention to quit. This indicates that preventive efforts focused on the less experienced and new CWP workers might have a greater effect than efforts targeted at the general population of CWP workers.

## Data Availability

The dataset presented in this article is not readily available due to privacy concerns. Requests to access the datasets should be directed to the corresponding author at kristel.h.nilsen@uit.no.

## Abbreviations

CWP: Child welfare and protection
ITQ: Intention to quit.
JD-R: Job demands and resources
